# Large genotype–phenotype study in carriers of D4Z4 borderline alleles provides guidance for facioscapulohumeral muscular dystrophy diagnosis

**DOI:** 10.1038/s41598-020-78578-7

**Published:** 2020-12-10

**Authors:** Giulia Ricci, Fabiano Mele, Monica Govi, Lucia Ruggiero, Francesco Sera, Liliana Vercelli, Cinzia Bettio, Lucio Santoro, Tiziana Mongini, Luisa Villa, Maurizio Moggio, Massimiliano Filosto, Marina Scarlato, Stefano C. Previtali, Silvia Maria Tripodi, Elena Pegoraro, Roberta Telese, Antonio Di Muzio, Carmelo Rodolico, Elisabetta Bucci, Giovanni Antonini, Maria Grazia D’Angelo, Angela Berardinelli, Lorenzo Maggi, Rachele Piras, Maria Antonietta Maioli, Gabriele Siciliano, Giuliano Tomelleri, Corrado Angelini, Rossella Tupler

**Affiliations:** 1 Department of Life Sciences, University of Modena and Reggio Emilia, Modena, Italy; 2 Department of Clinical and Experimental Medicine, Neurological Clinic, University of Pisa, Pisa, Italy; 3 Department of Neurosciences, Reproductive and Odontostomatological Sciences, University Federico II of Naples, Naples, Italy; 4 Department of Statistics, Computer Science and Applications “G. Parenti”, University of Florence, Florence, Italy; 5 Department of Neuroscience, Center for Neuromuscular Diseases, University of Turin, Turin, Italy; 6 Department of Biomedical, Metabolic and Neural Sciences, University of Modena and Reggio Emilia, Via G. Campi 287, 41125 Modena, Italy; 7 Center for Neuroscience and Neurotechnology, University of Modena and Reggio Emilia, Modena, Italy; 8 Neuromuscular Unit, Fondazione IRCCS Ca’ Granda Ospedale Maggiore Policlinico, Dino Ferrari Center, University of Milan, Milan, Italy; 9 Neurology Clinic, Spedali Civili Hospital, Brescia, Italy; 10 INSPE and Division of Neuroscience, IRCCS San Raffaele Scientific Institute, Milan, Italy; 11 Department of Neurosciences, University of Padua, Padua, Italy; 12 Center for Neuromuscular Disease, CeSI, University “G. D’Annunzio”, Chieti, Italy; 13 Department of Neurosciences, Policlinico “G. Martino”, University of Messina, Messina, Italy; 14 Department of Neuroscience, Mental Health and Sensory Organs, S. Andrea Hospital, University of Rome “La Sapienza”, Rome, Italy; 15 Department of Neurorehabilitation, IRCCS Eugenio Medea, Bosisio Parini, Italy; 16 Unit of Child Neurology and Psychiatry, IRCCS “C. Mondino” Foundation, Pavia, Italy; 17 IRCCS Foundation, C. Besta Neurological Institute, Milan, Italy; 18 ASL8, Centro Sclerosi Multipla, Cagliari, Italy; 19 IRCCS San Camillo, Venice, Italy; 20 Department of Molecular Cell and Cancer Biology, University of Massachusetts Medical School, Worcester, USA; 21 Li Weibo Institute for Rare Diseases Research at the University of Massachusetts Medical School, Worcester, USA

**Keywords:** Medical research, Molecular medicine, Neurology, Risk factors

## Abstract

Facioscapulohumeral muscular dystrophy (FSHD) is a myopathy with prevalence of 1 in 20,000. Almost all patients affected by FSHD carry deletions of an integral number of tandem 3.3 kilobase repeats, termed D4Z4, located on chromosome 4q35. Assessment of size of D4Z4 alleles is commonly used for FSHD diagnosis. However, the extended molecular testing has expanded the spectrum of clinical phenotypes. In particular, D4Z4 alleles with 9–10 repeat have been found in healthy individuals, in subjects with FSHD or affected by other myopathies. These findings weakened the strict relationship between observed phenotypes and their underlying genotypes, complicating the interpretation of molecular findings for diagnosis and genetic counseling. In light of the wide clinical variability detected in carriers of D4Z4 alleles with 9–10 repeats, we applied a standardized methodology, the Comprehensive Clinical Evaluation Form (CCEF), to describe and characterize the phenotype of 244 individuals carrying D4Z4 alleles with 9–10 repeats (134 index cases and 110 relatives). The study shows that 54.5% of index cases display a classical FSHD phenotype with typical facial and scapular muscle weakness, whereas 20.1% present incomplete phenotype with facial weakness or scapular girdle weakness, 6.7% display minor signs such as winged scapula or hyperCKemia, without functional motor impairment, and 18.7% of index cases show more complex phenotypes with atypical clinical features. Family studies revealed that 70.9% of relatives carrying 9–10 D4Z4 reduced alleles has no motor impairment, whereas a few relatives (10.0%) display a classical FSHD phenotype. Importantly all relatives of index cases with no FSHD phenotype were healthy carriers. These data establish the low penetrance of D4Z4 alleles with 9–10 repeats. We recommend the use of CCEF for the standardized clinical assessment integrated by family studies and further molecular investigation for appropriate diagnosis and genetic counseling. Especially in presence of atypical phenotypes and/or sporadic cases with all healthy relatives is not possible to perform conclusive diagnosis of FSHD, but all these cases need further studies for a proper diagnosis, to search novel causative genetic defects or investigate environmental factors or co-morbidities that may trigger the pathogenic process. These evidences are also fundamental for the stratification of patients eligible for clinical trials. Our work reinforces the value of large genotype–phenotype studies to define criteria for clinical practice and genetic counseling in rare diseases.

## Introduction

Facioscapulohumeral muscular dystrophy (FSHD, OMIM #158900) has prevalence of one in 8–20,000^1,2^. The disease is characterized by a peculiar distribution of muscle weakness affecting facial and shoulder girdle muscles. Abdominal muscles also become affected, leading to a characteristic hyperlordotic posture. The selective and precocious weakness of *tibialis anterior* muscle is typical at lower limb, eventually followed by proximal leg muscles involvement^3,4^.

Presently, FSHD diagnosis is based on molecular findings^5^. Two genetically distinct disease subtypes, FSHD1 and FSHD2, have been described up to now^6^. FSHD1 is associated with contractions of a polymorphic macrosatellite repeat on chromosome 4q35.2^7^. This region consists of tandemly arrayed 3.3 kb D4Z4 repeat elements ranging from 11 to > 100 repeat units in healthy subjects. Individuals displaying FSHD symptoms and carrying D4Z4 alleles with 10 or fewer repeat units are genetically defined as FSHD1^8^. They represent 95% of people with FSHD.

FSHD2 defines 5–10% of affected individuals carrying two D4Z4 arrays in the healthy range (> 10 repeat units). FSHD2 is defined as the cause of SMCHD1 mutation in OMIM and Gene Table (http://www.musclegenetable.fr). FSHD1 and FSHD2, considered clinically undistinguishable, are characterized by DNA hypomethylation of the 4q35 D4Z4 array^9^.

FSHD is characterized by reduced penetrance and wide variability in the clinical expression among patients and within families^10–13^. Indeed, the widespread use of molecular analysis to diagnose FSHD has revealed various phenotypes in subjects carrying D4Z4 alleles of reduced size, including atypical or incomplete phenotypes^12–19^. Furthermore, it has been observed that in the general population 3% of people carry D4Z4 array in the FSHD size range^20–22^. All this makes genotype–phenotype association difficult and hinders proper diagnosis, especially in presence of atypical clinical presentation^13^. Molecular factors such as level of D4Z4 methylation or mutations in other genes (i.e. *SMCHD1*or *DNMT3B* genes) have been recently added to the list of possible contributors to disease onset and modifiers of disease severity, and also to explain a digenic inheritance for FSHD^9,23–25^; even though more extended analysis suggest the limited contribution of these factors^26,27^. This clinical and molecular complexity has complicated FSHD diagnosis, clinical practice and genetic counseling. Based on the above considerations, how can we interpret and use the result of the FSHD molecular test?

Here, we investigate the occurrence of the FSHD classical phenotype in 134 index cases and 110 relatives carrying alleles with 9–10 D4Z4 repeat units from the Italian National Registry for FSHD (INRF). Alleles of this size, named borderline D4Z4 reduced alleles (bDRA), are considered the upper size of the diagnostic range and pose the major diagnostic challenges. In fact, a wide phenotypic spectrum including atypical or incomplete phenotypes with no affected relatives is frequently observed in subjects carrying a bDRA^28^, making the definition of a clear cut-off point problematic.

## Materials and methods

### Study design and participants

In this study, we enrolled subjects carrying a bDRA. Index cases were identified through the INRF considering a 9-year time-window (2008–2016). The INRF database contains clinical and molecular data of subjects examined by the Italian Clinical Network for FSHD (www.fshd.it)^29^. Out of 1340 index cases bearing a DRA with 1–10 repeats, we identified 166 subjects (14.6%) carrying a bDRA (Supplementary Fig. 1). Clinical and molecular analysis was extended to all available and willing to participate relatives. Phenotypic characterization was performed on 134 index cases and 110 relatives carrying a bDRA from 58 unrelated families. Seventy-six index cases did not have available relatives carrying a bDRA (Supplementary Fig 2).

### Availability of data

The dataset analyzed during the current study is available from the corresponding author upon request.

### Procedures

We used the Comprehensive Clinical Evaluation Form (CCEF) for the clinical characterization of each subject^30^. This evaluation protocol defines the severity of the motor impairment, and generates the FSHD score, which translates disability into a number. It ranges from zero, when no objective evidence of muscle functional impairment is present, to 15, when all the muscle groups tested are severely impaired (www.fshd.it)^31^. Subjects were further classified on the basis of the clinical signs considering typical and atypical features, as listed in the CCEF (Supplementary Fig. 3): (1) subjects presenting facial and scapular girdle muscle weakness typical of FSHD (category A, subcategories A1–A3), (2) subjects with muscle weakness limited to scapular girdle or facial muscles (category B, respectively subcategories B1 and B2), (3) asymptomatic without any functional motor impairment (FSHD score 0) or healthy subjects (category C, respectively subcategories C1 and C2), (4) subjects with myopathic phenotype presenting clinical features not consistent with FSHD canonical phenotype (D, subcategories D1, D2). Age at onset was estimated as self-reported anamnestic record^32^.

### Molecular characterization

DNA was prepared from isolated lymphocytes according to standard procedures. In brief, restriction endonuclease digestion of DNA was performed in agarose plugs with the appropriate restriction enzyme: *Eco*RI, *Eco*RI/*Bln*I. Digested DNA was separated by pulsed field gel electrophoresis (PFGE) in 1% agarose gels, as previously described^18^. Allele sizes were estimated by Southern hybridization with probe p13E-11 of 7 μg of *Eco*RI-digested, *Eco*RI/*Bln*I-digested genomic DNA extracted from peripheral blood lymphocytes, electrophoresed in a 0.4% agarose gel, for 62–64 h at 35 V, alongside an 8–48 kb marker (Bio-Rad). Participants carrying alleles of 36–41 kb (9–10 D4Z4 units) in size were included in the study. Within the European population, pathological D4Z4 contractions usually are associated with the permissive 4qA haplotype in the subtelomeric region of chromosome 4q. The qA polymorphism was assessed by *Hin*dIII digestion and hybridization with qA probe. Restriction fragments were detected by autoradiography or by using the Typhoon Trio system (GE Healthcare). To verify that the obtained shortened D4Z4 fragment on chromosome 4 has a causative 4qA haplotype, an additional *Hin*dIII Southern blot was performed as suggested for the molecular diagnosis of FSHD1^33^.

### Statistical analysis

We used descriptive statistics for quantitative variables (mean and standard deviation) and qualitative variables (relative frequencies). Associations between qualitative variables were assessed using chi-square test or Fisher exact test. Associations between clinical parameters were described and tested using Pearson r correlation coefficient. Associations between quantitative variables and qualitative variables were evaluated using *t* test or ANOVA.

### Ethics approval and consent to participate

The INRF database was approved by the Provincial Ethics Committee of Modena (2712/CE). Informed written consent was obtained from all study participants, in accordance with the ethical standards of the 1964 Declaration of Helsinki.

### Consent for publication

This manuscript does not contain any individual person’s data in any form. Each patient was identified by a unique alphanumeric identification code and all data were made anonymous.

## Results

### Different phenotypes of index cases carrying 9–10 DRA

**Figure 1:**
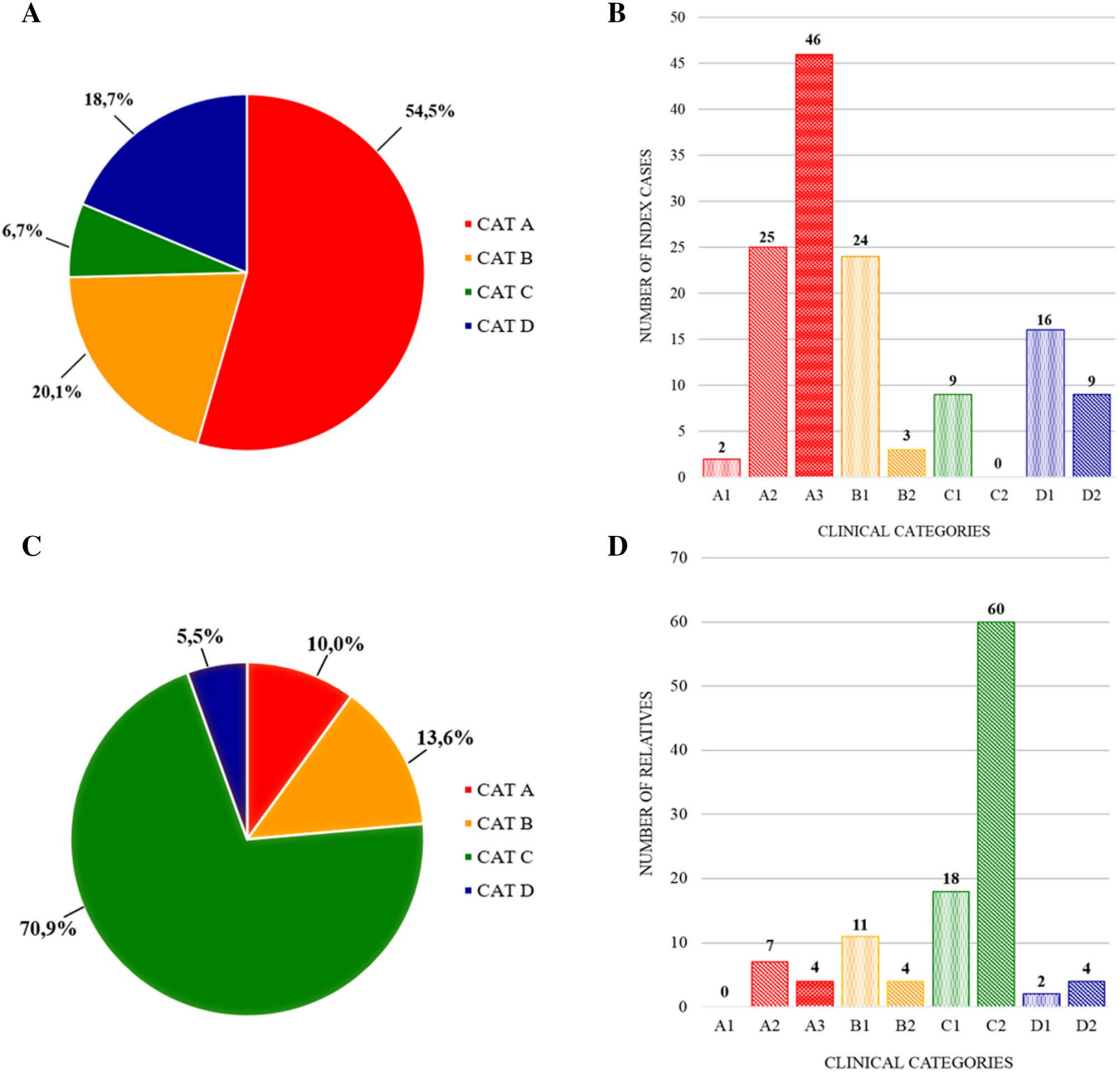
Phenotypic characterization of index cases and relatives carrying a bDRA on the basis of the CCEF categories. Distribution of clinical categories (**A**) and subcategories (**B**) among index cases. Distribution of clinical categories (**C**) and subcategories (**D**) among relatives.

The clinical characterization of 134 index cases carrying a bDRA (Table 1A) revealed their phenotypic heterogeneity (Fig. 1A). Seventy-three (54.5%) displayed the classic FSHD phenotype and were classified as category A. The remaining 61 index cases (45.5%) showed various phenotype: 27 presented muscle weakness limited to scapular girdle or facial muscles (20.1%), identified as category B, 25 subjects showing myopathic phenotypes with clinical features not consistent with FSHD (18.7%) were listed as category D, 9 subjects (6.7%) did not show motor impairment (FSHD score 0) and were classified as subcategory C1 for the presence of mild hyperCKemia and winged scapula.

**Table 1:** Distribution of clinical categories among subjects carrying a bDRA.

CCEF category (n)	Mean age at evaluation	Mean age at onset	Mean FSHD score
**a. Index cases 134 (84 M, 50 F)**			
A 73 (46 M, 27 F)	57.5 ± 15.8	32.9 ± 16.0	6.8 ± 3.0
B 27 (20 M, 7 F)	51.6 ± 15.1	35.3 ± 18.2	3.0 ± 1.8
C 9 (4 M, 5 F)	33.7 ± 20.9	–	–
D 25 (14 M, 11 F)	56.1 ± 13.0	38.2 ± 18.5	6.0 ± 3.4
**b. Relatives 110 (56 M, 54 F)**			
A 11 (3 M, 8 F)	55.2 ± 19.3	32.2 ± 17.6	5.5 ± 2.8
B 15 (12 M, 3 F)	51.7 ± 19.7	35.3 ± 17.0	2.5 ± 2.1
C 78 (39 M, 39 F)	42.0 ± 16.0	–	–
D 6 (2 M, 4 F)	57.0 ± 27.8	45.0 ± 36.1	5.3 ± 4.5

**Figure 2:**
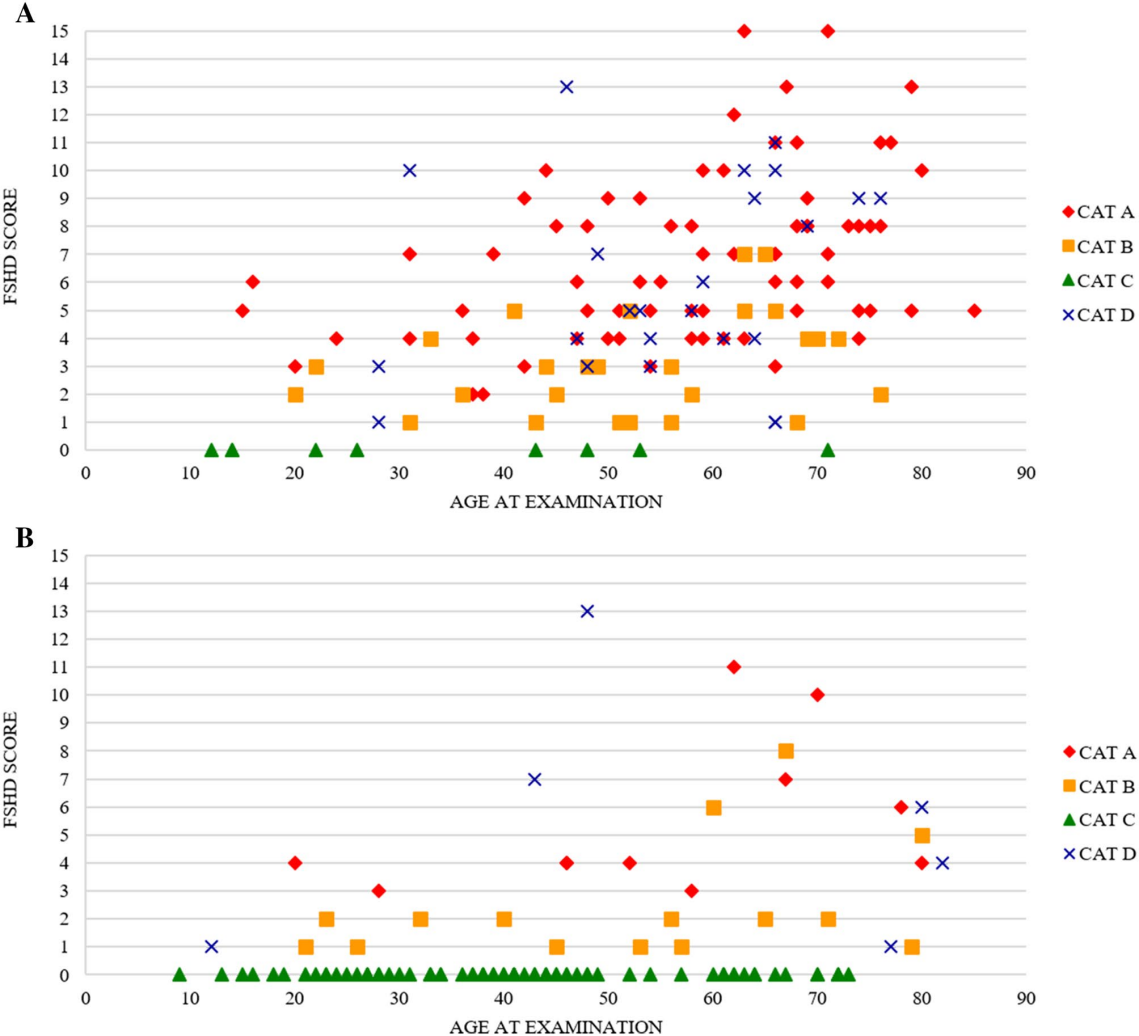
Distribution of clinical severity among subjects carrying a bDRA according to FSHD score and age at examination: index cases (**A**) and relatives (**B**). Clinical categories are described as follows red diamond (category A), yellow square (category B), green triangle (category C), blue cross (category D).

As expected for a slowly progressive disease as FSHD, we observed that there is a correlation between the FSHD score and age at examination or disease duration (Pearson coefficient equal to 0.37 and 0.36 respectively) in all patients falling in categories A, B, or D. We therefore evaluated whether clinical categories may represent different stages of disease progression that is pre-symptomatic carriers are included in category C, mildly affected individuals are in category B and fully manifesting subjects are in category A. If this is the case one would expect category C subjects to be the youngest and category A the oldest. Figure 2A shows that distribution of clinical categories is not influenced by the age at examination.

### Index cases with classic FSHD phenotype show a moderate-severe form of disease

Among the 73 index cases classified as category A, 2 presented severe facial weakness and were classified as subcategory A1, 25 showed the classical pattern of facial weakness (subcategory A2), 46 presented mild facial involvement (subcategory A3) (Fig. 1B). The mean age at onset of category A index cases was 32.9 ± 16.0, 53.4% of them reported disease onset after 30 years of age with the progressive development of a moderate-severe motor impairment (mean age at evaluation 57.5 ± 15.8, mean FSHD score 6.8 ± 3.0) (Table 1).

### Incomplete FSHD phenotype is associated with milder motor disability

Facial-sparing scapular myopathy is often detected in clinical practice and it must be distinguished from other forms of myopathy including scapular peroneal syndrome^34–36^. To further investigate this aspect, we compared the degree of muscle impairment of subcategory B1 and category A excluding the scoring of facial muscle weakness. As shown in Table 2, the index cases with facial-sparing phenotype classified as subcategory B1 had comparable mean age at evaluation and mean age at onset of category A index cases. Instead the average FSHD score assessed in category B1 subjects was significantly lower than the one detected in category A subjects (3.2 ± 1.8 versus 5.6 ± 2.8, *t* test *p* < 0.001). Thus, subcategory B1 patients have a milder clinical phenotype.

**Table 2:** Index cases with typical (category A) and facial-sparing (category B1) phenotype.

CCEF category (n)	Mean age at evaluation	Mean age at onset	FSHD score excluding facial scoring
A (73)	57.5 ± 17.8	32.9 ± 16.0	5.6 ± 2.8*
B1 (24)	51.6 ± 15.0	33.3 ± 17.1	3.2 ± 1.8*

*p < 0.001.

Three index cases, aged 31, 52 and 70 years, were classified as subcategory B2 showing facial weakness without scapular girdle involvement. They presented a mild motor impairment, FSHD score 1, 1, 4 respectively. Two of them had isolated weakness of facial muscles.

### Complex/atypical phenotypes in bDRA index cases

Out of 134 index cases, 25 index cases (18.7%) were classified as category D. Sixteen patients were identified as subcategory D1 for the presence of additional atypical features, more frequently including prevalent pelvic girdle weakness and axial involvement with bent spine and dropped head. Nine index cases (6.7%) presented phenotypes inconsistent with FSHD, such as isolated axial weakness (i.e. bent spine syndrome), or other clinical conditions described in Table 3; therefore they were classified as subcategory D2.

**Table 3:** Index cases with atypical clinical features (clinical category D).

Case	Sex	Age	Age at onset	Atypical phenotypic features	Family history	Clinical category	Other relatives with bDRA (category)
1	F	60	40	Axial involvement (bent syndrome), cardiac involvement	Negative	D2	
2	F	59	48	Pelvic limb girdle onset	Negative	D1	Daughter (C)
3	M	28	17	Recurrent myoglobinuria	Negative	D2	
4	F	64	25	Pelvic limb girdle onset, LGMD-like	Negative	D1	
5	F	66	50	Isolated pelvic girdle involvement	Negative	D2	Two sons (both C)
6	M	74	16	Dropped head	Negative	D1	
7	M	54	43	Pelvic limb girdle onset	Negative	D1	Three relatives (all C)
8	F	31	0	Congenital facio-brachio-crural hemiparesis	Negative	D2	Mother and maternal aunt (both C)
9	F	64	6	Prevailing pelvic girdle involvement	Negative	D1	
10	M	66	54	Axial involvement (bent syndrome)	Negative	D1	Son (C)
11	F	63	20	LGMD-like	Negative	D2	Brother and sister (both C)
12	M	69	55	Axial involvement	Negative	D2	
13	M	47	39	Early gastrocnemius atrophy and weakness	Negative	D1	
14	M	66	50	LGMD-like	Negative	D2	
15	M	66	66	Isolated pelvic girdle involvement	Negative	D2	Two sons (both C)
16	M	61	47	Prevailing axial involvement	Negative	D1	
17	F	49	43	LGMD-like	Negative	D1	Three relatives (C)
18	M	52	48	Dropped head	Negative	D1	
19	F	54	50	Bent syndrome	Negative	D2	
20	M	76	70	Axial involvement	Negative	D1	
21	F	53	41	LGMD-like	Positive	D1	
22	F	48	20	Diagnosis of myasthenia gravis	Positive	D1	Two sons (both C) and sister (A)
23	F	46	24	Prevailing pelvic girdle involvement	Negative	D1	
24	M	28	18	Blood CPK > 4 × normal value No winged scapula	Positive	D1	Father (B)
25	M	58	54	Prevailing pelvic girdle involvement	Negative	D1	

There was also the case of a woman (patient 22 in Table 3) that was classified as subcategory D1 because of the concomitant diagnosis of myasthenia gravis associated with abnormally elevated serum levels of acetylcholine receptor antibodies and thymoma. She was the only one among category D index cases who reported a positive family history for FSHD.

To date, all the others have not received any alternative diagnosis yet.

### Analysis of prognostic significance of bDRA in relatives

**Figure 3:**
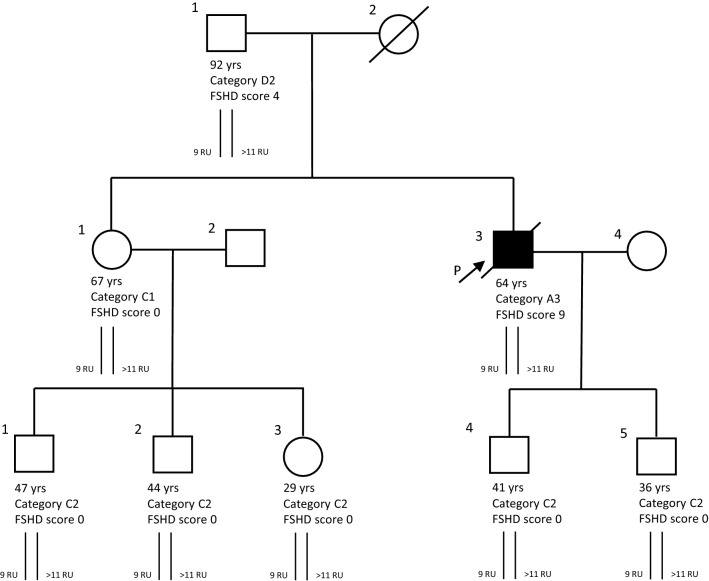
Pedigree Family 952. Age (years) at clinical evaluation, FSHD score (sc), CCEF clinical category, D4Z4 molecular haplotype are reported. Individuals I.1, II.1, II.3, III.1, III.2, III.3, III.4, III.5 carry one D4Z4 allele with 9 RU associated with the qA polymorphism. Individual II.3 developed FSHD at 45 years in the period following three cycles of chemotherapy.

We clinically evaluated 110 relatives carrying a bDRA (56 males, 54 females, mean age at evaluation 45.4 ± 18.2) from 58 unrelated families (Table 1b, Fig. 1C). Among them, 78 subjects (70.9%) did not show motor impairment (mean age at evaluation 42.0 ± 16.0) and were classified as category C (Fig. 1C), in particular 56 (54.5%) were completely normal at neurological examination (subcategory C2) and 22 (16.4%) showed minor signs (winged scapula and/or horizontal clavicles), without motor impairment (subcategory C1) (Fig. 1D). Eleven (10.0%) were identified as category A, 15(13.6%) as category B, 6 (5.5%) as category D and subcategorized as shown in Fig. 1D. Figure 2B shows the distribution of clinical categories among relatives according to age at examination and FSHD score. We observed non-penetrant carriers in all classes of age, accordingly with previous reports^12^. In one family we found that the index case developed FSHD at 45 years of age in the period following three cycles of chemotherapy because of non-Hodgkin lymphoma. In this family 5 relatives carrying the 9 U DRA were healthy whereas the 92-year-old father received FSHD score 4 at neurological examination for limb girdle weakness, therefore classified as category D2 (Fig. 3).

We evaluated the penetrance of bDRA in 12 families with at least 4 carriers. Table 4 summarizes our findings: the probands have different phenotypes. The overall penetrance of bDRA is 40%, ranging from 0 in one family, in which all subjects had no muscle impairment and were assessed as category C, to 100% in two families; in one of these two the proband was assessed as category A as well two relatives, one relative was category D2; in the other, the proband was category A, two relatives were assessed as category B1, one as D1.

**Table 4:** Penetrance of bDRA in families with 4 or more carriers.

Family ID	Subjects with DRA (n)	Clinical category		Penetrance (%)
		Proband	Relatives (n)	
FSHD 1639	4	C1	C2 (3)	0
FSHD 219	5	A3	C2 (4)	20
FSHD 1779	5	A2	C (4)	20
FSHD 1011	4	D1	C2 (3)	25
FSHD 1722	4	D1	C2 (3)	25
FSHD 952	7	A3	D2 (1) C2 (5)	28
FSHD 135	9	A2	A2 (2) B1 (1) C (5)	45
FSHD 1239	4	B1	B1 (1) C (2)	50
FSHD 348	5	B1	B1 (2) C (2)	60
FSHD 1855	5	A2	D1 (1) D2 (1) C (2)	60
FSHD 1624	4	A3	A2 (2) D2 (1)	100
FSHD 1971	4	A2	B1 (2) D1 (1)	100

### Considerations on genetic counseling in family with bDRA

**Figure 4:**
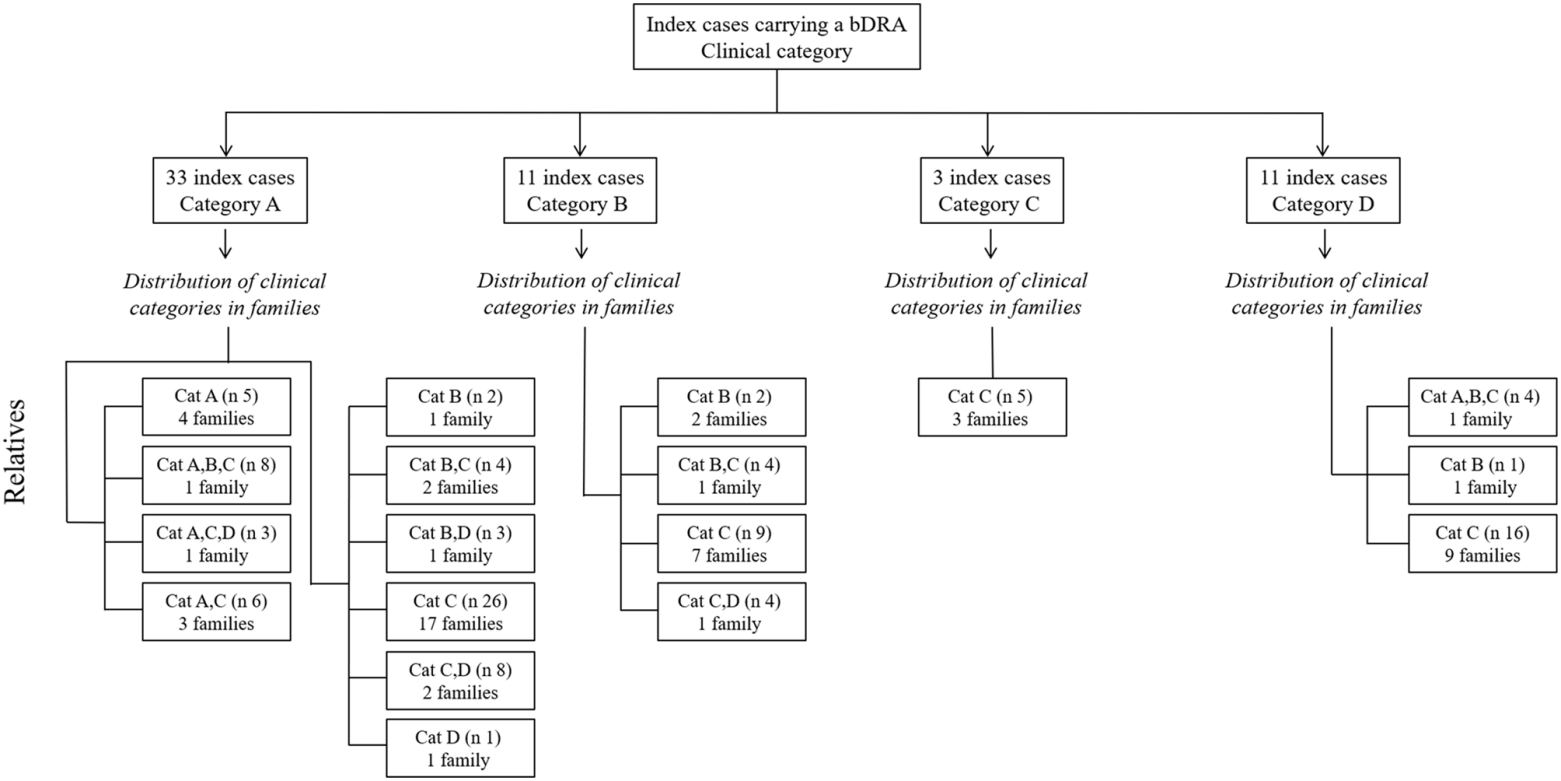
Distribution of the clinical categories observed among relatives in 58 families in which a bDRA segregates according to the clinical category of index cases. The total number of families in each group is indicated, as well as the total number of individuals examined (n).

We then evaluated the distribution of clinical phenotypes within families. Figure 4 shows that out of 58 families, in 10 (17.2%) there was at least one category A relative. In these families, the proband was classified as category A, with the exception of one family in which the index case was considered as D1 for the co-presence of myasthenia gravis, as described above. In 36 families (62%) all relatives were non-penetrant. In our large cohort, index cases assessed as category B did not have relatives that were classified as category A, instead in three families we observed only relatives of category B.

We then analyzed whether the clinical category of the index case can predict the phenotype observed in the relatives carrying the same bDRA. We subdivided the probands in two groups on the basis of the CCEF phenotype observed in their relatives. One group included 19 probands whose relatives had classical FSHD or incomplete phenotype (categories A and B); the other group included 39 probands whose relatives were healthy or had a complex phenotype (categories C and D). This analysis shows that the distribution of clinical categories among the 58 probands subdivided on the basis of the phenotypic categories of their relatives does not significantly differ (*p* value 0.161, Fisher exact test) as reported in Supplementary Table 1.

## Discussion

The present study substantiates the value of the clinical categories identified by the CCEF in response to the necessity of describing the different phenotypes of probands and their relatives and shows the possibility of stratifying clinical groups for clinical and molecular studies.

Through years molecular diagnostics in FSHD has faced several challenges mainly because of the wide clinical variability observed among patients and within families, the reduced penetrance of D4Z4 reduced alleles and their high frequency in the general population^12,14,15,22,37–41^. While there is a natural tendency to look for a relationship between number of repeat units and clinical severity, this approach has presented several flaws through years. Stratification of patients in clinical trials using the number of D4Z4 repeat units or methylation status has proven not to be accurate, suggesting that other disease-causing modifiers can modulate the clinical outcome^13,27^. Up to now, studies had involved a small number of patients with a higher D4Z4 alleles range^28,40,42^. This study selectively investigated the phenotype of the largest cohort of subjects with 9–10 D4Z4 repeats previously described, thus adding new data supporting clinical practice and genetic counseling.

Our study suggests that the clinical categories in adulthood do not represent different stages of disease course but identify specific phenotypes, as the distribution of clinical categories is not strictly influenced by the age at examination. In the group of bDRA carriers the majority of patients with a classic FSHD phenotype showed an adult-onset form of disease with a moderate-severe degree of muscle impairment. Remarkably, cases with facial-sparing phenotype, subcategory B1, had milder muscle impairment and have no relatives with classical FSHD phenotype. A similar observation has been recently reported in a cohort of Chinese patients and in FSHD patient population from the UK FSHD patient registry^36,43^. Notably, in our recently published 5-year follow-up study^44^ confirmed that clinical progression varies in people showing the different phenotypes described by the CCEF clinical categories. The distribution of clinical categories observed in subjects carrying a bDRA is also similar to those reported in our concomitant study conducted on cohort of 422 individuals carrying a DRA with 7 to 8 D4Z4 repeats from INFR^13^, thus highlighting that among carriers of D4Z4 reduced allele it is possible to recognize different clinical subgroups with distinct clinical features that need ad hoc studies.

In particular, the present study confirms the wide clinical variability among carriers of bDRA, highlighted by the fact that 46% of index cases do not show the classical FSHD phenotype with facial and shoulder girdle involvement. Remarkably, Table 3 shows that among atypical phenotypes, the prevalent clinical feature is the involvement of axial and pelvic muscles, which are features observed in several genetic and acquired myopathies. As an example, axial muscle weakness has been reported among carrier of DRA^45,46^. However, it is also known that several muscle diseases, such as inflammatory myopathies, limb-girdle muscular dystrophies, congenital myopathies or metabolic myopathies, can primarily affect the axial muscles and produce bent spine syndrome^47^.

Moreover, the finding of a family (Fig. 3) with several healthy carriers in which the only subject who developed after receiving anticancer treatments reinforces the idea that among carriers of bDRA disease may manifest itself through a multistep process. It is indeed possible that the bDRA constitutes a sensitizing condition that in presence of additional elements, genetic, epigenetic or environmental, favors the development of a myopathic phenotype affecting different muscles thus explaining the wide spectrum of clinical phenotypes.

Overall, this possibility is emphasized by the low penetrance of bDRA and by the fact that more than 70% of carrier relatives in our study show no motor impairment. In all these cases, family analysis (see example Fig. 3) adds crucial information.

Furthermore, as shown in Fig. 4, the analysis of intra-familial phenotypes and mode of inheritance can help to identify pedigrees in which alternative diagnosis must be considered or new genes can be searched.

Our study shows that in the group of carriers of bDRA the molecular marker is relevant neither for the diagnosis, all categories are represented, nor for the assessment of the genetic risk in relatives, overall 70.9% of relatives are healthy. Instead the distribution of the phenotypes within each family can provide information about the possible mechanism leading to disease. Genetic counseling should be guided by the extended clinical investigation of the proband’s family. Families in which the pedigree analysis suggests mendelial models of inheritance should be investigated to find candidate genes responsible for disease. In isolated cases one should consider additional factors contributing to disease development.

Overall, the evidences reported by the Italian Clinical Network for FSHD highlight the need to consider a standardized assessment for the best clinical management, for selection of patients in testing genetic modifiers and for trial readiness^48^.

## Conclusions

Since we found that in our healthy control population 3.7% of subjects carry a bDRA, the detection of it should be consider as a genetic susceptibility condition rather than a diagnostic marker. Accordingly, our large cohort study confirmed that the diagnostic value of bDRA is poor and, therefore, the recurrence risk cannot be estimated.

**Figure 5:**
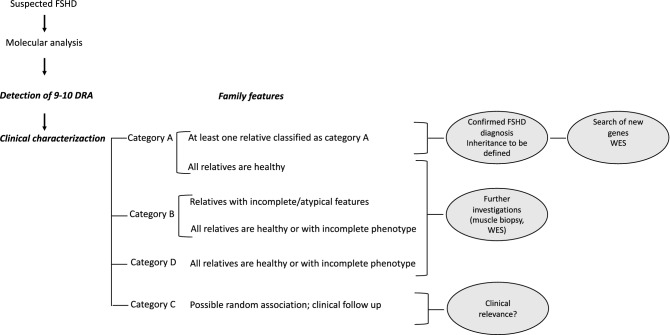
Proposal of a diagnostic flow chart for index cases with bDRA.

We therefore recommend diagnostic procedures and genetic counseling to be based on clinical data and family studies (Fig. 5). In families in which the index case and at least one affected relative show the classical FSHD phenotype, the diagnosis of FSHD can be performed and the mode of inheritance (autosomal dominant? recessive? X-linked?) should be investigated; these families can be selected to further studies in order to search for additional genes/modifier/causative factors. In presence of atypical phenotypes and/or isolated cases with all healthy relatives it is not possible to perform conclusive diagnosis of FSHD. All these cases need further investigation, such as muscle biopsy, for a proper diagnosis or to investigate environmental factors or co-morbidities that may trigger the pathogenic process (Fig. 5). Nowadays different high-throughput sequencing approaches, such as Whole Genome Sequencing or Whole Exome Sequencing, are available for family studies aimed at identifying genetic factors, including molecular mosaicism, contributing to the clinical phenotype.

Our recommendations constitute the basis for stratification and definition of eligibility criteria for trial readiness of cases in which a bDRA is detected.

## Supplementary information

Supplementary Figure 1. Absolute number of index cases collected by the Italian National Registry for FSHD. (INRF). Cases are distributed on the basis of the size of D4Z4 alleles reported as number of Repeat Units and the correspondent molecular weight as kilobases(kb).

Supplementary Figure 2. Selection of index cases and their relatives carrying a bDRA for genotype–phenotype correlation analysis.

Supplementary Figure 3. Clinical categories of CCEF (from reference [30]).

Supplementary Table 1. Distribution of probands on the basis of the clinical phenotype of relatives.

## Data Availability

The datasets used and/or analyzed during the current study are available from the corresponding author upon request.

## Abbreviations

FSHD: Facioscapulohumeral muscular dystrophy
FSHD1 and FSHD2: Facioscapulohumeral muscular dystrophy type 1 and type 2
CCEF: Comprehensive Clinical Evaluation Form
DRA: D4Z4 reduced alleles
bDRA: Borderline D4Z4 reduced alleles
INRF: Italian National Registry for FSHD
SMCHD1: Structural maintenance of chromosomes flexible hinge domain containing 1
DNMT3B: DNA methyltransferase 3 beta
