# Supplementary figures and images for: Large genotype–phenotype study in carriers of D4Z4 borderline alleles provides guidance for facioscapulohumeral muscular dystrophy diagnosis
Source: Sci Rep. 2020 Dec 10;10:21648. doi: 10.1038/s41598-020-78578-7 (PMC7730397; doi:10.1038/s41598-020-78578-7)

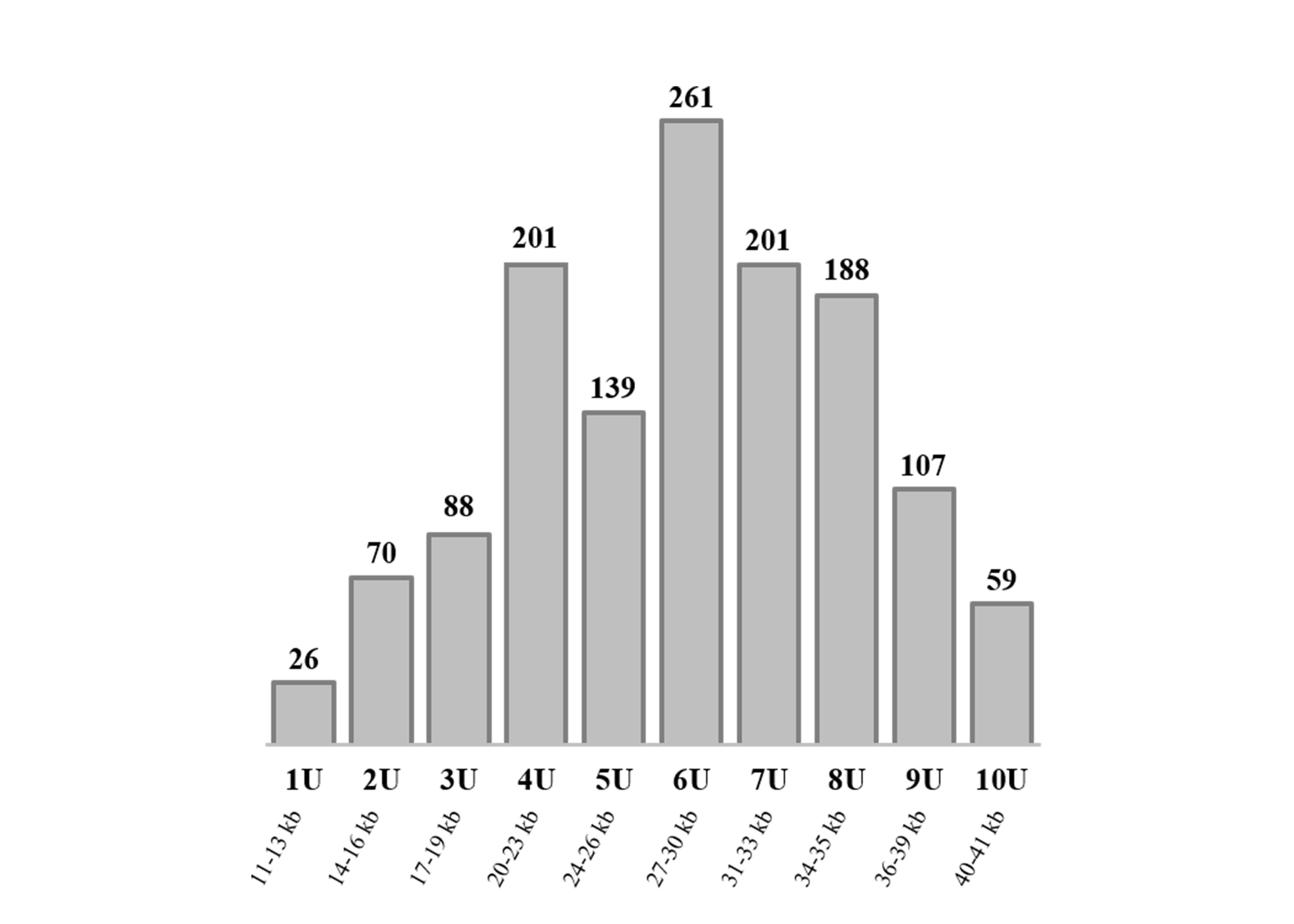

Supplement: Supplementary file 1 — Supplementary Figure 1. Absolute number of index cases collected by the Italian National Registry for FSHD. (INRF). Cases are distributed on the basis of the size of D4Z4 alleles reported as number of Repeat Units and the correspondent molecular weight as kilobases(kb). [file 41598_2020_78578_MOESM1_ESM.tiff]

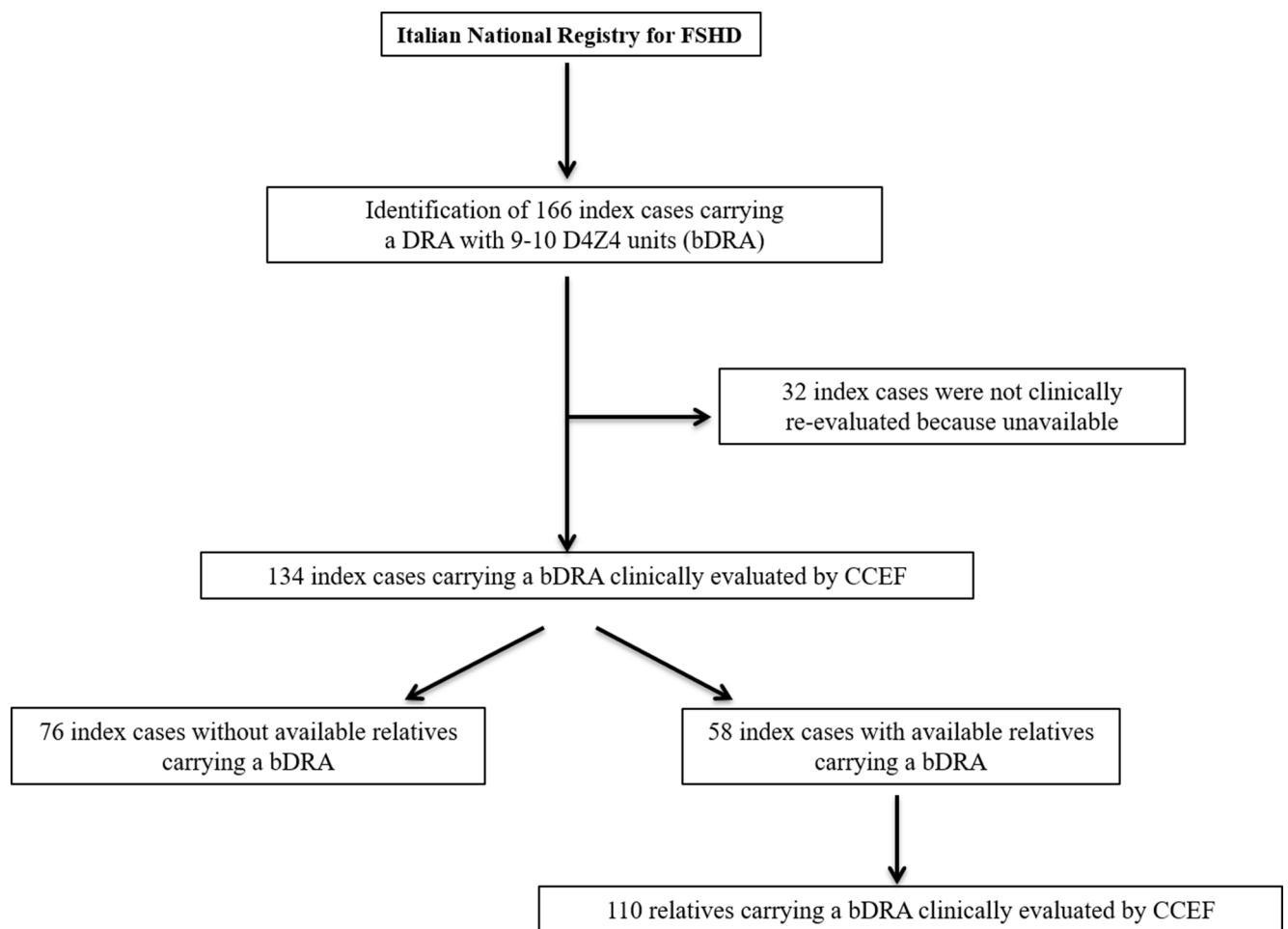

Supplement: Supplementary file 2 — Supplementary Figure 2. Selection of index cases and their relatives carrying a bDRA for genotype–phenotype correlation analysis. [file 41598_2020_78578_MOESM2_ESM.pdf]
