# Supplementary material for: Large genotype–phenotype study in carriers of D4Z4 borderline alleles provides guidance for facioscapulohumeral muscular dystrophy diagnosis
Source: Sci Rep. 2020 Dec 10;10:21648. doi: 10.1038/s41598-020-78578-7 (PMC7730397; doi:10.1038/s41598-020-78578-7)
Supplement: Supplementary file 3 — Supplementary Figure 3. Clinical categories of CCEF (from reference [30]). [file 41598_2020_78578_MOESM3_ESM.docx]

| CATEGORY A |
| --- |
| Category A1  Severe facial weakness (unable both to close eyes and to protrude lips) + impairment of upper limb abduction with winged scapula (scapular FSHD score ≥1) + absence of uncommon features  Category A2  Facial weakness (upper and lower facial weakness) + impairment of upper limb abduction with winged scapula (scapular FSHD score ≥1) + absence of uncommon features  Category A3  Facial weakness (upper or lower facial weakness) + impairment of upper limb abduction with winged scapula (scapular FSHD score ≥1) + absence of uncommon features |
| CATEGORY B |
| Category B1  Impairment of upper limb abduction with winged scapula (scapular FSHD score ≥1), no facial weakness + absence of uncommon features  Category B2  Facial weakness (facial FSHD score ≥1) + absence of uncommon features |
| CATEGORY C |
| Category C1  Subject with presence of at least one typical sign + FSHD score =0  Category C2  Subject without signs of muscle weakness + FSHD score =0 |
| CATEGORY D |
| Category D1  Subject fulfilling criteria of categories A1, A2, A3, B1, B2 + at least one uncommon feature  Category D2  -Subject fulfilling criteria of categories C1 or C2 + at least one uncommon feature  -Subject no fulfilling criteria of all above categories |
