# Supplementary material for: Large genotype–phenotype study in carriers of D4Z4 borderline alleles provides guidance for facioscapulohumeral muscular dystrophy diagnosis
Source: Sci Rep. 2020 Dec 10;10:21648. doi: 10.1038/s41598-020-78578-7 (PMC7730397; doi:10.1038/s41598-020-78578-7)
Supplement: Supplementary file 4 — Supplementary Table 1. Distribution of probands on the basis of the clinical phenotype of relatives. [file 41598_2020_78578_MOESM4_ESM.docx]

**Supplementary Table 1: Distribution of probands on the basis of the clinical phenotype of relatives**

| *Proband’s phenotype* | *Probands with relatives of A or B categories*  *n (%)* | *Probands with relatives of C or D categories*  *n (%)* |
| --- | --- | --- |
| Category A | 14 (73.7) | 19 (48.7) |
| Category B | 4 (21.1) | 7 (17.9) |
| Category C | 0 (0) | 3 (7.7) |
| Category D | 1 (5.3) | 10 (25.6) |
| Total | 19 | 39 |
| p value (Fisher exact test) 0.161 | | |
